# Array-based techniques for fingerprinting medicinal herbs

**DOI:** 10.1186/1749-8546-6-18

**Published:** 2011-05-18

**Authors:** Linhai Niu, Nitin Mantri, Chun Guang Li, Charlie Xue, Edwin Pang

**Affiliations:** 1 School of Applied Sciences, Health Innovations Research Institute, RMIT University, Melbourne, Victoria 3000, Australia; 2 Division of Chinese Medicine, School of Health Sciences, Health Innovations Research Institute, RMIT University, Melbourne, Victoria 3000, Australia

## Abstract

Poor quality control of medicinal herbs has led to instances of toxicity, poisoning and even deaths. The fundamental step in quality control of herbal medicine is accurate identification of herbs. Array-based techniques have recently been adapted to authenticate or identify herbal plants. This article reviews the current array-based techniques, *eg *oligonucleotides microarrays, gene-based probe microarrays, Suppression Subtractive Hybridization (SSH)-based arrays, Diversity Array Technology (DArT) and Subtracted Diversity Array (SDA). We further compare these techniques according to important parameters such as markers, polymorphism rates, restriction enzymes and sample type. The applicability of the array-based methods for fingerprinting depends on the availability of genomics and genetics of the species to be fingerprinted. For the species with few genome sequence information but high polymorphism rates, SDA techniques are particularly recommended because they require less labour and lower material cost.

## Background

Bioactive compounds in certain medicinal herbs affect cell communication and signalling [1], induce inflammatory responses [2] and help prevent diseases [3]. Chinese medicinal herbs such as ginseng (*Panax ginseng*), *Danshen *(*Salvia miltiorrhiza*), Korean Mint (*Agastache rugosa*), Chinese motherwort (*Leonurus japonicus*) are globally recognized for treating human disorders. Currently, the global market for medicinal herbs currently is valued over $60 billion a year and growing at an annual rate of 6.4% [4].

Development and acceptance of herbal medicine are hindered by misidentification and adulteration of medicinal herbs which may lead to loss of therapeutic potency and potential intoxication [5]. Authentication of medicinal herbs ensures their therapeutic potency.

Morphological and histological methods, which have been used for authentication, are subjective and ineffective [6]. Chromatographic fingerprinting (*eg *HPLC) can be affected by the variations in growing conditions, harvesting periods and processing methods of the herbs [7]. Genomic tools were developed to fingerprint herbal plants as genomic information is more specific and does not readily change with environmental factors. Polymerase chain reaction (PCR)-based techniques, *eg *random amplified polymorphic DNA (RAPD) [8-10], amplified fragment length polymorphism (AFLP) [11] and sequencing-based techniques based on species-specific sequences, *eg *internal transcribed spacer (ITS) [12], have also been used to identify herbal species. PCR-based methods are limited by agarose gel electrophoresis which is time consuming and not feasible for large scale genotyping operations [13]. Moreover, some PCR-based methods such as microsatellites and sequence characterised amplified regions (SCAR) require prior sequence information and may not be suitable for fingerprinting the species with poor genomic resources [13,14].

DNA microarrays were used to identify medicinal herbs by detecting the hybridisation between fluorescent targets and probes spotted on the microarray [15,16]. In comparison with PCR-based techniques, array-based techniques enable a larger number of DNA probes (or targets) to hybridise with labelled targets (or probes); thus they are more accurate, less time consuming and labour intensive. Array-based techniques include sequence-dependent microarrays and sequence-independent microarrays. Sequence-dependent microarrays are subdivided by type into oligonucleotide microarrays [17,18] and gene-based probe microarrays [6]; sequence-independent arrays are subdivided into Diversity Array Technology (DArT™) [19], Subtracted Diversity Array (SDA) [20] and Suppression Subtractive Hybridisation (SSH)-based arrays [14,21]. The salient features of these techniques will be reviewed in subsequent sections of this article.

Array-based fingerprinting has not been thoroughly reviewed in literature. For instance, while nine PCR-based methods used for identifying Chinese medicinal materials were reviewed, array-based fingerprinting was only discussed briefly [22]. Another review covered recent patents on DNA extraction, DNA amplification, the generation of DNA sequences and fingerprints and high-throughput authentication methods [23]. Two other reviews discussed various fingerprinting techniques for the authentication of herbal species [24,25]. These reviews, however, did not provide details about the latest array-based techniques like the SDA technique.

The present article reviews sequence-dependent and sequence-independent array techniques for fingerprinting medicinal herbs [6,14,15,17-21,24,26,27] (Additional file 1) and compares these techniques according to important parameters such as sample conditions, markers, polymorphism rates, restriction enzymes and hybridisation techniques (Table 1).

**Table 1:** Comparison of the array-based techniques used for fingerprinting medicinal plants

	Oligonucleotide microarrays	Gene-probe based microarrays	DArT™	SDA	SSH-based arrays
Sequence information required	Yes	Yes	No	No	No
Restriction enzymes used	None	None	Yes [usually one rare cutter (*Pst*I) and one frequent cutter (*Taq*I/*Bst*NI/*Hae*III)]	Yes [two frequent cutters (*Hae*III and *Alu*I)]	Yes [one frequent cutter (*Rsa*I)]
Subtraction Suppression Hybridisation required	No	No	No	Yes (one)	Yes (multiple pairwise)
Probe preparation	Chemical synthesis	PCR amplified products	Selective amplified products of the digested DNA fragments	Subtracted DNA fragments	Subtracted/Restriction digested DNA fragments
Target preparation	PCR amplified products	PCR amplified products	Selective amplified products of the digested DNA fragments	Restriction digested DNA fragments	the other choice against probe preparation
Dye system used*	Single-dye	Single-dye	Single/Dual-dye	Single-dye	Single/Dual-dye
Polymorphism rate	Species specific	Species specific	Up to 27%	Up to 68%	Up to 42.4%

Note: * The 'Dye system used' row reveals the dye systems used by researchers to fingerprint medicinal plants.

In practise, single/dual dyes can be used on all microarray platforms.

### Sequence-dependent microarrays

**Figure 1:**
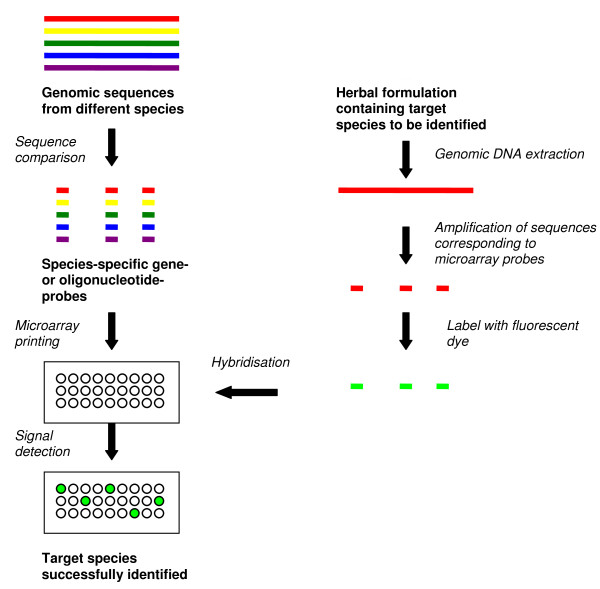
**Method of manufacturing and using oligonucleotide or gene-probe based microarray for fingerprinting herbal plants**. The species-specific gene or oligonucleotide probes can either be PCR amplified or chemically synthesized for microarray printing. Fingerprinting herbal species with a single dye system is shown in the figure; however, it is possible to use a dual-dye system where one sample can be a reference and other a test sample, or both samples can be test samples.

This type of microarrays is dependent on availability of genomic sequence information for the species of interest. Genomic sequences are compared for identification of non-redundant sequences unique to a particular species. Hundreds and thousands of such species-specific sequences can be spotted on a single microarray to help identify herbal tissue from a constituent herbal species of a complex herbal formulation (Figure 1).

#### Oligonucleotide microarrays

In an oligonucleotide microarray, unique 25 to 60 nucleotide species-specific probes are printed on a glass or quartz platform. The probe sequences are either from coding or non-coding regions of a plant's genome. Each oligonucleotide microarray may potentially have probes from hundreds of herbal plant species. A herbal plant species to be authenticated is referred to as a target species. Genomic DNA is extracted from the target species; the sequences corresponding to the oligonucleotide probes are amplified, fluorescently labelled and hybridised onto the oligonucleotide array under highly-stringent conditions. The hybridisation is then quantified by laser-based detection for determination of relative abundance of target species-specific sequences on the array (Figure 1). This technique was successfully applied in the identification of eight toxic medicinal plant species using oligonucleotide probes based on spacer region between the coding regions of the 5S rRNA gene [17]. In another study, 33 species-specific oligonucleotide probes based on the 18S rRNA gene of 13 *Panax *species were successfully used to differentiate closely related *Panax *species [18].

#### Gene-based probe microarrays

This type of microarrays is similar to oligonucleotide microarrays except that these arrays are made of unique sequences from coding regions of the plant genomes and the probe size can potentially span the whole gene length (between 0.5 and 1.5 kb). For example, the internal transcribed spacer (ITS) ribosomal DNA sequences, which are usually species-specific, are amplified from different herbal species and subsequently spotted as probes on glass slides. Extraction of genomic DNA from target species, hybridisation and detection steps are similar to oligonucleotide arrays (Figure 1). However, compared to oligonucleotide microarrays, a single gene fragment is used as a probe on gene-based probe microarrays instead of several oligos from a gene sequence. These microarrays may also be more specific since larger DNA fragments are used as probes. A study using this technique obtained distinctive signals for the five medicinal *Dendrobium *species listed in the Chinese Pharmacopoeia [6]. This type of microarray was sensitive enough for detecting the presence of *Dendrobium nobile *in a Chinese medicinal formulation containing nine herbal components [6]. While both oligonucleotide and gene-based microarrays can differentiate herbal plants at the species level, they may not be appropriate for fingerprinting herbal species with poor genomic information as both types of techniques require prior sequence information for primer or oligo design.

### Sequence-independent arrays

An alternative to sequence-dependent microarrays is sequence-independent microarrays constructed by reduction of genome complexity.

#### Diversity arrays technology (DArT™)

**Figure 2:**
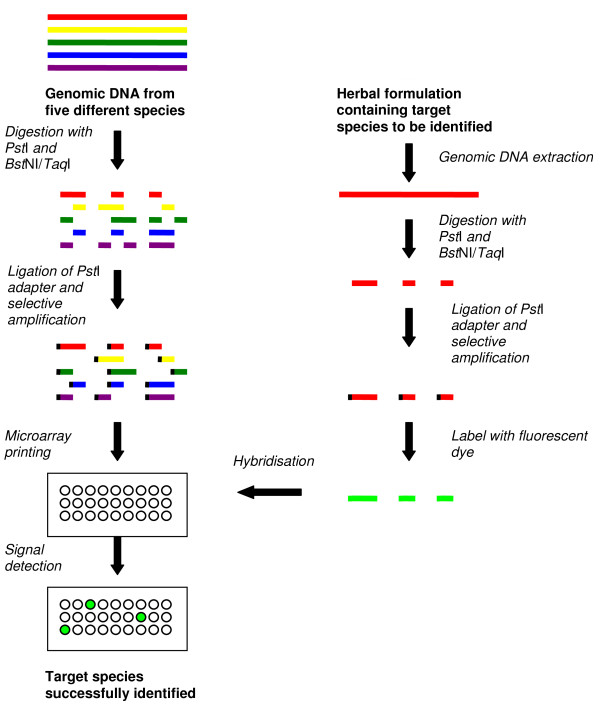
**Method of manufacturing a microarray with diversity array technology (DArT™) and using it for fingerprinting herbal plants**. Fingerprinting herbal species using a single dye system is shown in the figure; however, it is possible to use a dual-dye system where one sample can be a reference and other a test sample, or both samples can be test samples. The method for construction of a DArT™ based microarray for five species is shown in the figure; however, the number of species used could vary according to the experimental design.

DArT™, a sequence-independent microarray originally developed for authenticating rice [13], has been widely used to investigate the genetics of polyploid species such as wheat [28] and sugarcane [29]. This technique uses a combination of restriction endonucleases, usually *Pst*I along with a frequent cutter such as *Alu*I, *Bst*NI, or *Taq*I to produce genomic representations of genomic DNA samples from the species to be fingerprinted. A *Pst*I adapter with an overhang is subsequently ligated to the restriction fragments which then are selectively PCR amplified and cloned into vectors. These representative clones are spotted on microarray glass slides as probes. When an unknown specimen is to be identified, the target DNA is digested with the same restriction enzymes, ligated with *Pst*I adapters, PCR amplified, labelled with fluorescent dyes and hybridised onto the DArT array (Figure 2). This technique reduces the genomic complexity by 100- to 1000-fold of the original genomic DNA pool and allows fingerprinting of any organism or a group of organisms belonging to the same genome pool from which the microarray was developed [30]. DArT was used to fingerprint *Eucalyptus grandis*, a medicinal plant [19].

Compared with the sequence-dependent microarrays, the DArT™ is labour intensive due to the requirement of restriction digestion, adaptor ligation and selective amplification (Table 1). These steps increase the level of technical difficulty especially when a large number of species are fingerprinted. Moreover, comparatively low level of polymorphism rates (between 3% and 27%) were reported in the previous DArT™ studies, which is a potential weakness of this technique [13,28,29,31,32].

#### Suppression subtractive hybridization (SSH)-based arrays

**Figure 3:**
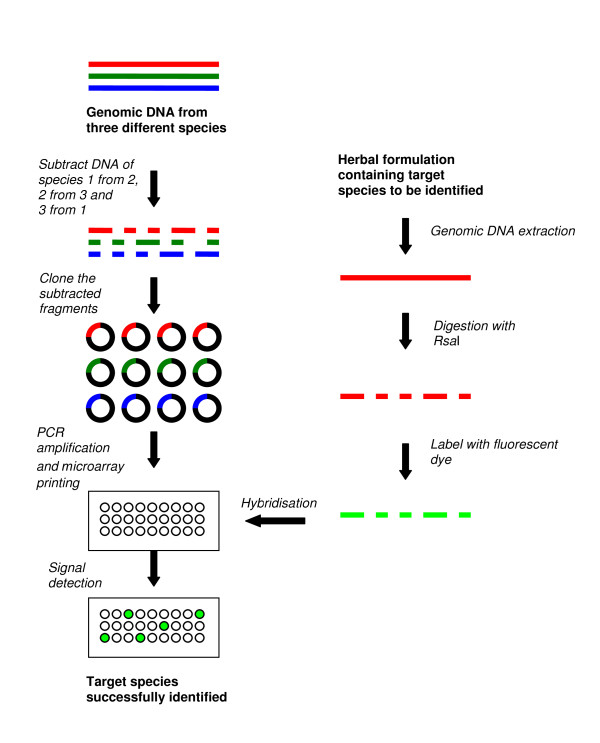
**Method of manufacturing a suppression subtractive hybridization-based microarray and using it for fingerprinting herbal plants**. Fingerprinting herbal species using a single dye system is shown in the figure; however, it is possible to use a dual-dye system where one sample can be a reference and other a test sample, or both samples can be test samples.

SSH procedure was first commercialised by Clontech^® ^(USA) through the development of PCR-Select™ cDNA subtraction kit to enrich for rare sequences over 1,000-fold using subtractive hybridization. In this method, the cDNA containing specific (differentially expressed) genes is referred to as 'tester' and the reference cDNA as 'driver'. The tester and driver cDNAs are separately digested with a frequent cutting restriction enzyme, namely *Rsa*I to generate shorter blunt-end fragments. The tester cDNA is subsequently divided into half and ligated with two different sets of adapters. The *Rsa*I digested driver cDNA is then added in excess to both the tester cDNA pools and two different hybridisation reactions are performed to selectively amplify the differentially expressed cDNA sequences from the tester pool. In one of the first uses of this technique, testis-specific cDNA fragments were extracted and used as probes to identify homologous sequences in a human Y chromosome cosmid library [33]. SSH has since been widely used for gene expression studies and modified for DNA fingerprinting [14,20,21,34]. In general, pair-wise DNA subtractions are performed between species to be fingerprinted and species-specific sequences are spotted on microarray slides as probes (Figure 3). With SSH-based microarray, five species of the genus *Dendrobium *viz., namely *D. aurantiacum *Kerr, *D. officinale *Kimura et Migo, *D. nobile *Lindl., *D. chrysotoxum *Lindl. and *D. fimbriatum *Hook were successfully fingerprinted [14,21].

However, this method is costly and labour intensive, and perhaps impractical for fingerprinting a large number of species. In the study by Li *et al*. [14], four subtractions were performed to fingerprint only six species of *Dendrobium*.

#### Subtracted diversity array (SDA)

**Figure 4:**
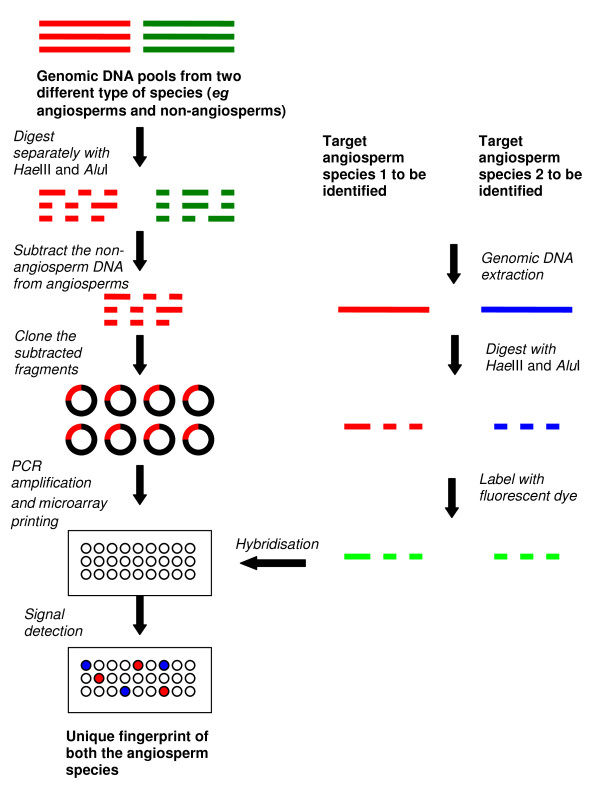
**Method of manufacturing a subtracted diversity array and using it for fingerprinting herbal plants**. The method shown in the figure is for subtraction of non-angiosperm DNA from angiosperm DNA which is a broad subtraction. This subtraction was capable of fingerprinting species from different angiosperm clades and orders with high (68%) polymorphism [20] but showed lower (10-22%) polymorphism when fingerprinting closely related species [36]. However, genomic DNA pool of species belonging to a particular family or order can be subtracted from genomic DNA pool of other family or order for a closer subtraction to obtain high polymorphism for closely related species [Mantri, unpublished data]. It is possible to use a single/dual-dye system where one sample can be a reference and other a test sample, or both samples can be test samples as shown in the figure.

SDA, a novel microarray, was constructed based on a modified SSH-based array technique [34]. Instead of making pair-wise subtractions between the species to be fingerprinted [14], Jayasinghe *et al*. pooled genomic DNA from 49 representative angiosperm species and subtracted this DNA from pooled genomic DNA of five representative non-angiosperm species to extract angiosperm-specific DNA fragments [34]. The angiosperm-specific DNA fragments were printed on microarray glass slides and used as probes to fingerprint species from different angiosperm clades (Figure 4). SDA successfully discriminated species from all the six main clades of APG II classification system [35] and correctly clustered nine species at the family level [20]. SDA was used to discriminate dried herbal samples including a few closely related species, *eg Magnolia denudata *and *Magnolis biondii*, *Panax ginseng *and *Panax quinquefolius *[36]. Furthermore, this technique was sensitive enough to identify a 10% deliberate contamination of *Panax quinquefolius *DNA in pure *Panax ginseng *DNA [36]. SDA may be suitable for detecting DNA polymorphisms as it is cost-effective compared with DArT™ and SSH-based techniques for fingerprinting a large number of samples.

### Comparison of array-based techniques

#### Type of herbal plant samples

Oligonucleotide microarrays and gene-based probe microarrays use fresh and dried materials as samples, SSH-based arrays and DArT™ use only fresh samples while SDA fingerprints dried and fresh herbal plant materials. However, fingerprinting dried herbal materials is more difficult than fresh samples possibly because highly degraded DNA obtained from dried samples may have lower copy number of or less unique sequences/genes, thus reducing the number of polymorphic sequences, subsequently decreasing polymorphism rate and increasing fingerprinting difficulty. As medicinal herbs are usually sold in dried or powdered form, arrays capable of identifying dried samples may be more useful.

#### Restriction enzymes

Sequence-dependent microarrays use amplified products of species-specific sequences as probes and do not require restriction enzymes. On the other hand, in sequence-independent arrays, using appropriate restriction enzymes to generate sufficient number of polymorphic sequences is critical for the fingerprinting. For this reason, some DArT™ studies initially compared restriction enzyme sets [29,32], which is a time- and cost- consuming process. By contrast, SSH-based arrays and SDA do not require enzyme comparisons and only use frequent cutting enzymes as compared to DArT™. Frequent cutter(s) used in SDA (*Alu*I and *Hae*III) and SSH-based (*Rsa*I) arrays recognize 4 bp sequences and be beneficial for target preparation in comparison with rare (6 bp) cutters (*Pst*I; *Eco*RI) used in DArT™. Frequent cutters generate more fragments of smaller sizes than restriction enzymes recognizing 6 bp sequences. Longer fragments have less potential of generating polymorphic sequences than shorter ones as there are less selective nucleotides for selective amplification. This may explain why SDA generated higher polymorphism compared with previous DArT™ studies [20]. Moreover, restriction enzyme combination resulting in more complex genomic sequences is likely to generate more markers and reduce redundancy. Combination of frequent cutters, namely *Hae*III and *Alu*I may be used in future studies with DArT™.

#### Markers

Generation of molecular markers is a critical step for fingerprinting studies. Usually, sequence-dependent microarrays generate probes by amplifying the regions of species-specific nuclear or chloroplast genes. For instance, the species-specific oligonucleotide probes amplified from the *5S ribosomal RNA *gene of 19 herbal plants were used to fingerprint these herbs [15]. Furthermore, 23 bp to 26 bp sequences from the nuclear *18S ribosomal RNA *gene of 13 *Panax *species were employed as the species-specific probes to identify each individual species [18]. While these microarrays are cheap and not labour intensive, large number of PCR amplifications used in these methods may lead to artefacts thus affecting the fingerprinting results. Moreover, as closely related species have been found to possess identical sequence at the same loci [24], probes generated in those arrays may be insufficient for bar-coding purposes of all herbal plants. Therefore, sequence-independent arrays with multiple markers have recently been widely used to fingerprint herbal plants [19,20].

Sequencing of polymorphic markers from sequence-independent arrays has identified gene- and retroelement-based sequences. For instance, the sequences of many features on a DArT™ were revealed to match genes such as 'GGPP synthase' and possible retrotransposons [27]. Sequences of many probes on SDA have also been reported to match 'retroelements', 'genes' and 'putative uncharacterized proteins' [20]; however, the markers used to fingerprint plant species may have been different as different restriction enzymes were used in these studies. These restriction enzymes recognize different sequences and produce markers of varied lengths. For example, probes between 83 bp to 453 bp were used as markers in a SSH-based array [37] whereas markers ranging from 147 bp to 880 bp were used in a SDA [20] and markers with average length of 563 bp have also been reported [38].

Since a sufficient number of unique polymorphic markers is critical for generating reliable results, detecting redundancy level of those markers is a crucial step for sequence-independent arrays. Based on a literature search only five of eleven DArT™ studies reported the redundancy level, with redundancy levels being between 14% and 56%. Differences in redundancy levels may be attributed to different restriction enzymes and target/probe preparation methods used in these studies.

#### Polymorphism rates

Polymorphism rate refers to the percentage of polymorphic features (that discriminate between species) out of the total number tested. Polymorphism rates obtained with sequence-dependent microarrays cannot be directly compared with those of sequence-independent arrays as these arrays use different methodologies for target/probe preparation. Markers of sequence-dependent microarrays are designed or synthesised based on species-specific sequences. Thus, the polymorphic probes used may be sufficient for discriminating the species being fingerprinted, resulting in a high polymorphism rate. By contrast, markers of sequence-independent arrays are prepared from whole genomic DNA. For instance, markers of a few SSH-based arrays and SDA are generated based on subtracted genomic DNA or DNA pool whereas those of DArT™ are also prepared from genomic DNA pool. Fragments produced are cloned and PCR-amplified to generate probes. As the identity of these fragments is not known when they are spotted on the microarray, many of these probes are expected to be the same (redundant) thus reducing the polymorphic frequency.

Various polymorphism rates have been reported for sequence-independent arrays. In general, SSH-based arrays generated higher polymorphism rates than DArT™. For instance, a polymorphism rate of 42.4% was reported for a SSH-based array fingerprinting six *Dendrobium *species [14]. This number is higher than the polymorphism rate of 3 to 27% reported in DArT™ studies [13,19,29,32]. The possible reason is that the common sequences between testers and drivers were removed by SSH, thereby enriching the probe library with polymorphic sequences for the species of interest. Compared with SSH-based arrays, SDA showed a higher polymorphism rate of 68% when used to fingerprint medicinal plant species representing six different clades of the flowering plants [20]. This may be attributed to the wide subtraction of 49 angiosperm genomic DNA from five non-angiosperm genomic DNA performed during the development of SDA, as a comparison to the close pair-wise subtraction of species from the same genera performed for SSH-based array construction. Moreover, the species that were fingerprinted with SDA were significantly different from each other (belonging to six different clades) compared to those fingerprinted with SSH (belonging to the same genera). This argument is supported by low polymorphism rates of 22.3% and 10.5% obtained from fingerprinting (with SDA) closely related species, namely *Magnolia biondii *and *Magnolia denudata*, *Panax ginseng *and *Panax quinquefolius *respectively [36]. To overcome this, Mantri *et al*. performed a closer subtraction by subtracting genomic DNA of non-asterid species (non-asterid angiosperms and non-angiosperms) from genomic DNA of asterid species to fingerprint herbal plants from the asterid clade of plants. A polymorphism rate of 50% was obtained with this array to fingerprint 25 Asterid species from 20 families [Mantri, unpublished data].

Polymorphism rates obtained with array-based techniques for fingerprinting may also be affected by different methods of data analysis used for defining positive features. A less stringent threshold for positive spots may improve the sensitivity but can decrease the polymorphism rate of the experimental system. Thresholds used in previous sequence-independent array studies are not suitable for direct comparison because these studies used different labelling methods or thresholds to score the ratios. For instance, previous SDA studies used a threshold of 2.0 (signal ≥2 background) to define 'positive' features (features considered to show true signal) [20] while the DArT™ studies subtracted background from the signal to call 'positive' features [19]. Moreover, DArT™ studies used either a single-dye system involving Cy3 [19] or a dual-dye dye system using Cy3 with Cy5/FAM [28,29] to label the targets and the ratio of signal intensities between samples to score features. By contrast, a single-dye system using Cy3 to label targets and signal to background ratio within the sample was used to score features in the SDA studies [20]. Furthermore, compared to SDA and DArT™, SSH-based arrays used DIG to prepare targets for investigating the relationship of *Dendrobium *species and did not assess the spot intensities with laser-based scanning [14,21]. In SSH-based arrays, the ratio of signal intensity of one spot for two species is the signal intensity for one species divided by that for the other [14], which was used to replace the Cy3/Cy5 ratio to score the spots. Consequently, valid comparisons cannot be made between the methods to define the 'positive' features in different microarray studies.

## Discussion

The choice among these methods mainly depends on the genomics and genetics of the species to be fingerprinted. Sequence-dependent microarrays are fast and cheap, and capable of fingerprinting the species with sequence information available in the existing databases. In contrast, sequence-independent arrays are laborious and costly but suitable for identifying a large number of species which lack sequence information in the existing databases. Further, the sequence-independent arrays are less affected by the artefacts caused by large number of PCR amplifications during target preparation. SDA is advantageous over SSH-based arrays as SDA does not require multiple SSH for probe preparation. Further, SDA is also advantageous over DArT™ as SDA does not require adapter ligation and selective amplification for target preparation. As SDA is also sensitive enough for fingerprinting dried herbal samples, its use in fingerprinting of herbal plants is more versatile.

## Conclusion

The applicability of the array-based methods for fingerprinting depends on the availability of genomics and genetics of the species to be fingerprinted. For the species with few genome sequence information but high polymorphism rates, SDA techniques are particularly recommended because they require less labour and lower material cost.

## Abbreviations

AFLP: Amplified Fragment Length Polymorphism; cDNA: complementary DNA (deoxyribonucleic acid); Cy3: cyanine 3; Cy5: cyanine 5; DArT: Diversity Array Technology; DIG: Digoxigenin; DNA: Deoxyribonucleic acid; FAM: carboxyfluorescein; HPLC: High-performance liquid chromatography; ITS: Internal transcribed spacer; PCR: Polymerase chain reaction; RAPD: Random Amplified Polymorphic DNA; rRNA: ribosomal RNA (ribonucleic acid); SCAR: Sequence Characterised Amplified Regions; SDA: Subtracted Diversity Array; SSH: Suppression Subtractive Hybridization;

## Competing interests

The authors declare that they have no competing interests.

## Authors' contributions

NM and LN share equal first authorship and wrote the article with inputs from EP, CGL and CX. NM prepared the figures. All authors read and approved the final version of the manuscript.

## Supplementary Material

Additional file 1 **Summary of array-based methods for the studies of herbal plants**. The different array-based method used for fingerprinting medicinal plants are compared based on array method, kind of tissue used for DNA extraction, and substrate/platform used for microarray printing. The species that were fingerprinted and results obtained are highlighted. Click here for file
