# Supplementary material for: Array-based techniques for fingerprinting medicinal herbs
Source: Chin Med. 2011 May 18;6:18. doi: 10.1186/1749-8546-6-18 (PMC3121662; doi:10.1186/1749-8546-6-18)
Supplement: Additional file 1 — Summary of array-based methods for the studies of herbal plants. The different array-based method used for fingerprinting medicinal plants are compared based on array method, kind of tissue used for DNA extraction, and substrate/platform used for microarray printing. The species that were fingerprinted and results obtained are highlighted. [file 1749-8546-6-18-S1.DOC]

Additional file 1 - A summary of array-based methods used on the studies of herbal plants.

| **Array method** | **Species assessed** | **Sources of DNA** | **Substrate** | **Probes based on** | **Results** |
| --- | --- | --- | --- | --- | --- |
| Oligonucleotide microarray | *Allocasia nacrorrhiza*, *Datura innoxia*, *D. metel*,  *D. tatula*, *Pinellia cordata*, *P. ternata*,  *P. pedatisecta* and *Typhonium giganteum*. | Dried materials and fresh leaves | Silicon-based microarray | Spacer region of 5S-rRNA gene | *D. innoxia*, *D. metel* and *T. giganteum* were differentiated based on the differences of the hybridizations [17]. |
| Gene-based probe microarray | *Dendrobium chrysanthum, D. chrysotoxum,*  *D. crystallinum, D. densiflorum, D. falconeri,*  *D. fimbriatum, D. jenkinsii, D. lindleyi,*  *D. loddigesii, D. lohohense, D. moniliforme,*  *D. moschatum, D. nobile, D. pendulum,*  *D. primulinum.* | Fresh, medicinal formulation; leaves, stems | Microarray (glass) | Internal transcribed spacer 2 gene | Using the fluorescence-labeled ITS2 sequences as probes, the presence of *D. nobile* in a Chinese medicinal formulation containing nine herbal components was detected [6]. |
| Suppression Subtraction Hybridization-based array | *Dendrobium auriantiacum, D. nobile*  *D. chrysotoxum, D. fimbriatum and*  *D. officinale*. | Stems of commercially available  samples | Array (nylon) | Fragments from pair-wise subtraction of genomic DNA | Fourteen species-specific probes from five closely related *Dendrobium* species were used on the array. Various commercial *Dendrobium* samples and unrelated samples were successfully identified [27]. |
| Suppression Subtraction Hybridization-based array | *Dendrobium aurantiacum* Kerr, *D. officinale Kimura* et Migo, *D.nobile* Lindl., *D. chrysotoxum* Lindl. and *D. fimbriatum* Hook. | Fresh leaves | Array (nylon) | Fragments from pair-wise subtraction of genomic DNA | 72 samples of *Dendrobrium* spp. containing 21 samples of *D.officinale* Kimura et Migo, 11 samples of *D.chrysotoxum* Lindl. and 40 samples of other *Dendrobrium* spp. were screened using this array. This array could successfully identify the species used to generate the probes [21]. |
| Diversity Array Technology | *Eucalyptus grandis* | Fresh leaves | Microarray (glass) | *Pst*I digested genomic DNA | This array was prepared using a partial genomic library from total genomic DNA of 23 *E. grandis* trees, of which 22 were full siblings. 27% of the 384 fragments screened were found to be polymorphic and allowed identification of all the 17 full-sibling individuals tested [19]. |
| Oligonucleotide array | *Aconitum carmichaeli, A. pendulum, Alocasia macrorrhiza, Corton tiglium, Datura inoxia, D. metel, D. tatula, Dysosma versipellis, Euphorbia kansui, Hyoscyamus niger, Pinellia cordata, P. pedatisecta, P. ternate, Rhododendron molle, Stellera chamaejasme, Strychnos nux-vomica, Typhonium divaricatum, T. giganteum*. | Fresh leaves | Silicon-based microarray | Spacer region of 5S-rRNA gene | Multiple toxic plant species were successfully identified by parallel genotyping. *Datura inoxia, D. metel, D. tatula* were identified based on the differences in fluorescent intensities [15]. |
| Oligonucleotide array | *Aconitum napellus* Herb., *Arabidopsis thaliana* L., *A. absinthium* L., *A. vulgaris* L., *Atropa belladonna* L., *Capsicum annuum var.* *glabriusculum* L. (Dunal) Heiser & *Pickersgill, Caulophyllum thalictroides* L. Michx., *Citrus aurantium* L., *Datura metel* L., *Digitalis lanata* Ehrh., *Echinacea angustifolia*, DC., *Ephedra viridis* Coville, *Glycyrrhiza uralensis* Fisch. ex DC., *Hypericum perforatum* L., *Lawsonia inermis* L., *Lobelia inflata* L., *Mentha pulegium* L., *Symphytum officinale* L., *Tanacetum vulgare* L., *Teucrium canadense* L., *T. chamaedrys* L., *Tussilago farfara* L. | Fresh leaves | Microarray (glass) | Cytochrome P450 gene | The genes for cytochrome P450 enzymes were cloned and sequenced for probes design in MLPA assays for identification. The probes can detect the presence of their cognate genomic DNA [26]. |
| Suppression Subtraction Hybridization-based array | *Dendrobium aurantiacum* Kerr, *D. officinale* Kimura et Migo, *D. nobile* Lindl., *D. chrysotoxum* Lindl., *D. fimbriatum* Hook*.* and *D. densiflorum* Lindl. et Wall. | Fresh leaves | Array (nylon) | Fragments from pair-wise subtraction of genomic DNA | A dendrogram of the relatedness of six *Dendrobium* species was produced according to their polymorphic profiles. The results revealed that the SSH-based array was effective for profiling genomic DNA polymorphisms and dendrograms [14]. |
| Subtracted Diversity Array | A population of 28 angiosperm species (including 25 medicinal herbs) representing the six main clades in angiosperms | Fresh leaves | Microarray (glass) | Fragments from subtracting pooled genomic DNA | Pooled genomic DNA of 5 non-angiosperm species was subtracted from pooled genomic DNA of 49 angiosperm species to obtain 376 probes. Species representing the six angiosperm clades (Asterids, Rosids, Caryophyllids, Ranunculids, Monocots and Eumagnoliids) could be differentiated using the SDA. A polymorphism rate of 68% was obtained for the probes used [24]. |
| Oligonucleotide microarray | *P. ginseng* C. A. Meyer, *P. japonicus* C. A. Meyer (Japanese ginseng), *P. quinquefolius* L. (American ginseng), *P. notoginseng* (Burk.) F. H. Chen, *P. japonicus* C. A. Meyer var. *angustifolius* (Seem.) C. Y. Wu et Feng, *P. stipuleanatus* H. T. Tsai et K. M. Feng, *P. pseudoginseng* Wall. | Dried leaf or roots | Microarray (glass) | 18S rRNA gene | 33 probes corresponding to the species-specific nucleotide substitutions observed at 11 sites in the 18S rRNA gene sequence were used on the array. This array allowed successful authentication of all the *Panax* plants, drugs and derived health foods tested [18]. |
| Subtracted Diversity Array | 34 species belonging to six angiosperm clades and seven different families | Fresh leaves | Microarray (glass) | Fragments from subtracting pooled genomic DNA | This array could correctly genotype species that were not used for initial DNA pooling to generate probes. All species tested correctly clustered at the family level, but minor discrepancies were observed when the fingerprinting was performed at the species level [20]. |
